# Radiation Induces Pulmonary Fibrosis by Promoting the Fibrogenic Differentiation of Alveolar Stem Cells

**DOI:** 10.1155/2020/6312053

**Published:** 2020-09-29

**Authors:** Lu-Kai Wang, Tsai-Jung Wu, Ji-Hong Hong, Fang-Hsin Chen, John Yu, Chun-Chieh Wang

**Affiliations:** ^1^Radiation Biology Core Laboratory, Institute for Radiological Research, Chang Gung University/Chang Gung Memorial Hospital, Linkou, Taoyuan, Taiwan; ^2^Institute of Stem Cell and Translational Cancer Research, Chang Gung Memorial Hospital, Linkou, Taoyuan, Taiwan; ^3^Department of Radiation Oncology, Chang Gung Memorial Hospital, Linkou, Taoyuan, Taiwan; ^4^Department of Medical Imaging and Radiological Sciences, Chang Gung University, Linkou, Taoyuan, Taiwan; ^5^Radiation Biology Research Center, Institute for Radiological Research, Chang Gung University/Chang Gung Memorial Hospital, Linkou, Taoyuan, Taiwan

## Abstract

The lung is a radiosensitive organ, which imposes limits on the therapeutic dose in thoracic radiotherapy. Irradiated alveolar epithelial cells promote radiation-related pneumonitis and fibrosis. However, the role of lung stem cells (LSCs) in the development of radiation-induced lung injury is still unclear. In this study, we found that both LSCs and LSC-derived type II alveolar epithelial cells (AECII) can repair radiation-induced DNA double-strand breaks, but the irradiated LSCs underwent growth arrest and cell differentiation faster than the irradiated AECII cells. Moreover, radiation drove LSCs to fibrosis as shown with the elevated levels of markers for epithelial-mesenchymal transition and myofibroblast (*α*-smooth muscle actin (*α*-SMA)) differentiation in *in vitro* and *ex vivo* studies. Increased gene expressions of connective tissue growth factor and *α*-SMA were found in both irradiated LSCs and alveolar cells, suggesting that radiation could induce the fibrogenic differentiation of LSCs. Irradiated LSCs showed an increase in the expression of surfactant protein C (SP-C), the AECII cell marker, and *α*-SMA, and irradiated AECII cells expressed SP-C and *α*-SMA. These results indicated that radiation induced LSCs to differentiate into myofibroblasts and AECII cells; then, AECII cells differentiated further into either myofibroblasts or type I alveolar epithelial cells (AECI). In conclusion, our results revealed that LSCs are sensitive to radiation-induced cell damage and may be involved in radiation-induced lung fibrosis.

## 1. Introduction

Radiotherapy is a mainstay among treatments of thoracic malignancies such as lung cancer and esophageal cancer. However, pulmonary damages after high-dose radiation result in radiation pneumonitis in the early stages and pulmonary fibrosis later on [1]. Although numerous studies have been performed and it is widely recognized that cell death and inflammation play important roles in these processes [2], the exact cellular and molecular mechanisms underlying them are not fully elucidated. After irradiation, the injured airway epithelial cells undergo apoptosis and secrete cytokines and growth factors that recruit immune cells and alter the microenvironment. These inflammatory responses in the pneumonitis stage, which promote the maturation of fibroblasts and excess deposition of extracellular matrix, can subsequently develop into fibrosis [3, 4].

At the cellular and tissue levels, the alveolar epithelium is a major part of lung tissue and is composed of two types of epithelial cells: alveolar type I (AECI) cells and alveolar type II (AECII) cells. AECI cells cover >90% of the alveolar surface responsible for gas exchange, while AECII cells secrete surfactant for the maintenance of alveolar integrity and serve as progenitor cells for producing AECI cells. Recent studies suggest that terminally differentiated AECI cells undergo apoptosis for several months in response to ionizing radiation, which induces pulmonary pneumonitis [2, 5]. When AECI cells are destroyed, AECII cells increase proliferation and differentiate into AECI cells to reestablish the alveolar epithelium. On the other hand, irradiated AECII cells may contribute to the development of fibrosis [5–7]. Previous studies also indicated that rat AECII cells undergo epithelial-to-mesenchymal transition (EMT) through ERK/GSK3*β*/snail signaling after radiation treatment [7]. The process of EMT results in a loss of cell-cell junctions and epithelial polarity, downregulation of epithelial marker expression, such as E-cadherin, and upregulation of mesenchymal markers such as *α*-smooth muscle actin (*α*-SMA), fibroblast-specific protein-1 (FSP-1), and vimentin [5].

During normal tissue turnover or injury, stem cells are essential for the maintenance of homeostasis and repair of adult tissues. Lung tissue may be capable of self-renewal and differentiation by resident stem/progenitor cells to form various cell compartments following lung injury [8]. Many lung stem/progenitor cells (LSCs) have been reported and are potentially useful in regenerative therapy such as repair of damaged lung tissue in patients [9–12]. Previous studies have demonstrated that the Oct-4^+^ LSCs residing in the terminal bronchiolar region of the neonatal lungs are capable of being induced to differentiate into AECII and AECI cells [13]. Using a glycoproteomic strategy, we have identified surface markers for the prospective isolation of the CD45^−^CD54^+^CD157^+^ LSCs. These cells have the ability not only for self-renewal and differentiation *in vitro* but also for lung repair *in vivo* [14]. However, the effects of ionizing radiation on these CD45^−^CD54^+^CD157^+^ LSCs have not been investigated. Here, we demonstrated that these LSCs are more sensitive to radiation damage than their differentiated alveolar cells. In addition, using the fibrosis PCR array and immunostaining analyses, we showed that these irradiated LSCs underwent AECII and myofibroblast differentiation after irradiation and were involved in the fibrogenic response. Nintedanib, a tyrosine kinase inhibitor, is currently used to reduce the rate of decline in lung function in patients with idiopathic pulmonary fibrosis [15]. A single published study implied that nintedanib has antifibrotic activity after partial lung irradiation in mouse models; however, this cannot be monitored by the computed tomography imaging [16]. Tissue repair and airway remodeling involving the differentiation of LSCs are critical to the maintenance of lung homeostasis. The characterization of the radiation response of LSCs and their differentiated alveolar cells used in the present study is a critical approach to better define and understand the pathophysiology of fibrosis. Moreover, using *in vitro* cultured stem cells and differentiated cells of the lung may provide an easy-to-follow and less time-consuming platform for drug screening and pave the way for tissue engineering and stem cell therapy in the radiation research.

## 2. Materials and Methods

### 2.1. Mice and Irradiation

CD-1 (ICR) mice were purchased from BioLasco (Taiwan). Radiation was delivered using a 6 MV X-ray linear accelerator in the Proton and Radiation Therapy Center, Chang Gung Memorial Hospital, Linkou, Taiwan. For *in vitro* experiments, cells (density: 2.5 × 10^4^ cells/cm^2^) were exposed to 2, 4, or 8 Gy. For *ex vivo* experiments, neonatal CD-1 mice were treated with or without 8 or 15 Gy whole-body irradiation.

### 2.2. *In Vitro* Cell Culture

Primary lung stem cell (LSC) culture was performed as previously described [14]. LSCs were isolated from neonatal CD-1 mice by FACS sorting using phycoerythrin- (PE-) conjugated anti-CD157 (BioLegend, CA, USA), fluorescein isothiocyanate- (FITC-) conjugated anti-CD54 (BD Biosciences, CA, USA), and allophycocyanin- (APC-) conjugated anti-CD45 (eBioscience, CA, USA) antibodies. Isolated CD45^−^CD54^+^CD157^+^ cells or irradiated cells were maintained in Dulbecco's Modified Eagle's Medium (DMEM) supplemented with 10% FBS, 1% insulin–transferrin–selenium (ITS), and 1 ng/ml epidermal growth factors (EGF) (all obtained from Thermo Fisher Scientific, CA, USA) through several passages in a collagen I-coated plate. To conduct differentiation studies, the attached LSCs were incubated in MCDB-201 medium (Sigma-Aldrich, MO, USA) supplemented with 1% FBS, 1% ITS, and 10 ng/ml EGF for 7 or 14 days to induce AECII or AECI cells.

To determine the fibrogenic effect of transforming growth factor beta (TGF-*β*) and connective tissue growth factor (CTGF) (PeproTech, NJ, USA), LSCs (2.5 × 10^4^ cells/cm^2^) in 12-well culture plates were treated with TGF-*β* (5 ng/ml) or CTGF (50 ng/ml) for 3 days.

### 2.3. Immunofluorescence Staining and Quantification

Briefly, irradiated cells were washed, fixed in 4% paraformaldehyde/phosphate-buffered saline (PBS), and then blocked with 3% bovine serum albumin (BSA) in PBS for 30 min. Cells were incubated with primary antibodies at 4°C overnight. The following antibodies were used: anti-CD157 (BD Pharmingen, CA, USA); antiprosurfactant protein C (SP-C) (Millipore, CA, USA); antipodoplanin, also known as T1 alpha (T1*α*) (Santa Cruz Biotechnology, CA, USA); anti-*α*-smooth muscle actin (*α*-SMA) (DAKO, CA, USA); or antifibroblast-specific protein-1 (FSP1) (Merck, MA, USA). Cells were washed and incubated for 1 hour at room temperature with Alexa Fluor 488 donkey anti-mouse IgG or Alexa Fluor 594 donkey anti-rabbit IgG antibodies (Thermo Fisher Scientific) and then counterstained with DAPI (Invitrogen, CA, USA). Images were obtained using a Nikon Fluorescence Microscope (Nikon Instruments, NY, USA). For DNA repair assay, cells were fixed with methanol for 10 minutes followed by immunofluorescent analysis. For overnight incubation, the primary and secondary antibodies used were anti-phospho-histone H2AX (*γ*-H2AX) (Cell Signaling Technology, MA, USA) and Alexa Fluor 488 goat anti-rabbit IgG (Invitrogen), respectively. The cells were embedded with the ProLong Gold Antifade Mountant with DAPI. All slides were analyzed using a Nikon Fluorescence Microscope. The number of nuclear foci per cell was counted in 75 AECI cells and 150 LSCs or AECII cells using the FociCounter software (http://focicounter.sourceforge.net/index.html).

### 2.4. RNA Isolation and Quantitative Real-Time Reverse Transcription PCR

To perform the Mouse Fibrosis PCR Array analysis (Qiagen, CA, USA), total RNA was extracted from LSCs with or without irradiation using a TRIzol solution (Invitrogen) according to the manufacturer's instructions. The total RNA was then reverse-transcribed into cDNA using the RT^2^ first strand kit (Qiagen). Quantitative real-time RT-PCR (Q-PCR) was performed in triplicate using a reaction mixture containing RT^2^ SYBR Green qPCR Master Mix and 500 ng RNA in a CFX connect real-time PCR detection system (BIO-RAD, CA, USA). Alternatively, the total RNA was reverse-transcribed to cDNA using SuperScript III Reverse Transcriptase (Invitrogen). Q-PCR was performed to examine the expression of fibrosis genes. A mixture containing SYBR Green PCR Master Mix (Qiagen), 50 ng cDNA, and gene-specific forward and reverse primers (Supplementary table 1) was reacted in a CFX connect real-time PCR detection system. The relative expression levels of the target cDNA were calculated after normalizing the intensity of target cDNA to the intensity of *β*-actin in the Mouse Fibrosis PCR Array analysis or GAPDH in the gene expression examination.

### 2.5. Lung Differentiation Platform

A lung differentiation platform was established based on the colony picking of LSCs that induced differentiation with some modifications as described previously [13]. LSCs were seeded on one well of 2-well culture insert (Ibidi, Germany) in collagen I-coated *μ*-Slide (Ibidi) at 1 × 10^4^ cells per well. After the undifferentiated LSCs form an optically confluent monolayer, the culture insert was removed, and cells were maintained in DMEM supplemented with 10% FBS. After 5 days of culture, the cells at the edge region were migrated out and appeared as thinly spread flattened cell clusters. These cells received 4 Gy irradiation followed by treatment with or without nintedanib (1 *μ*M) (Boehringer Ingelheim, Germany), and the cell population for differentiated LSCs was examined using immunofluorescence staining at day 5.

## 3. Results

### 3.1. LSCs Are More Radiosensitive than Differentiated Alveolar Cells

**Figure 1:**
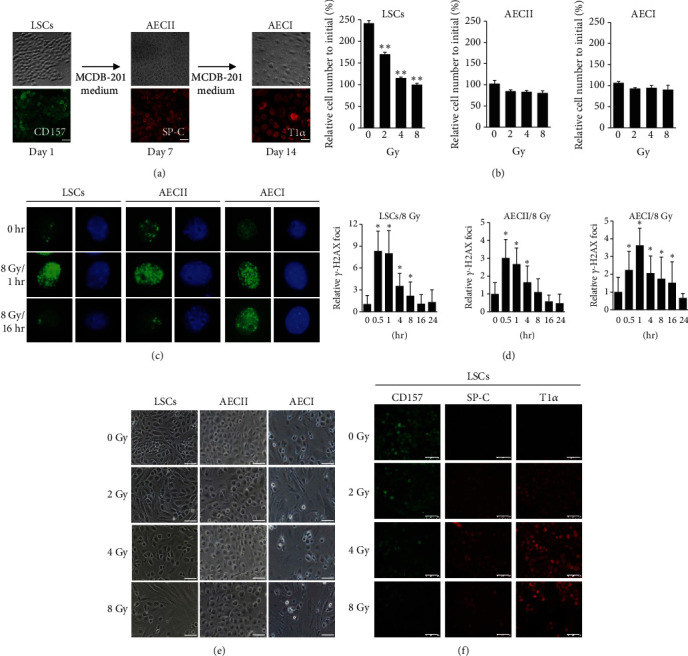
The lung cell differentiation and the effects of radiation exposure on LSCs and alveolar cells. (a) Workflow and time line of *in vitro* lung cell differentiation experiments. LSCs were isolated from neonatal ICR mice and then sequentially differentiated into alveolar cells by culture with MCDB-201 medium. LSCs, AECII, and AECI cells were examined through immunostaining with anti-CD157, anti-pro-SP-C, and anti-T1*α* antibodies. Scale bars, 100 *μ*m. (b) Cell numbers of irradiated LSCs and alveolar cells were examined at day 3 postirradiation. The results are represented as mean ± SD (*n* = 3, ^∗^*p* < 0.05) relative to initial cell number. (c) Immunostaining of *γ*-H2AX expression (green, left panels) and nuclear morphology (blue, right panels) in LSCs and alveolar cells that received 8 Gy at 1 h and 16 h. (d) The lung cells repair radiation-induced DNA double-strand breaks as determined by nuclear foci formation of *γ*-H2AX. The nuclear *γ*-H2AX foci per cell are means ± SD from LSCs and AECII cells (*n* > 100) or AECI cells (*n* > 75) relative to the initial sample. (e) The cell morphology of irradiated lung cells at day 3 postirradiation. Scale bars, 100 *μ*m. (f) The cell differentiation of irradiated lung stem cells was examined at day 3 postirradiation by immunostaining with specific protein markers CD157, SP-C, or T1*α*. Scale bars, 100 *μ*m.

In the present study, we succeeded in using the newly identified surface markers CD157, together with CD45 and CD54 to prospectively isolate LSCs by FACS analysis [14]. When cultured in DMEM growth medium, these isolated LSCs can proliferate and maintain the cobblestone epithelial morphology (Figure 1(a)). In contrast, these isolated LSCs grown in MCDB-201 differentiation medium showed that the cell shape became flattened at day 7 and expressed surfactant protein C (SP-C, AECII marker) (Figure 1(a)). An extension of the incubation to 14 days led to further flattening and thinly enlargement of the cells that expressed podoplanin (T1*α*, AECI marker) (Figure 1(a)). Therefore, these CD45^−^CD54^+^CD157^+^ LSCs display the capacity for self-renewal and differentiation into AECII cells and AECI cells in a sequential manner following induction signals in the culture medium [14]. To investigate the effect of radiation exposure in normal lung tissue, we irradiated the respective cells (LSCs, AECII, and AECI cells) with different doses of X-rays (0, 2, 4, and 8 Gy) to probe their sensitivity to radiation. After irradiation, the culture media in each cell type were replaced with DMEM growth medium immediately. On day 3 postradiation of LSCs, the cell numbers were assessed and found to decrease, in a dose-dependent manner (Figure 1(b)). In contrast, exposure to radiation had no apparent effect on the number of AECII and AECI cells (Figure 1(b)). These results suggest that LSCs have more capacity for active proliferation and are more radiosensitive to radiation damage than their differentiated alveolar cells.

To evaluate DNA damage and repair capacity in the irradiated lung cells, the DNA double-strand breaks were assessed by counting the number of phosphorylated histone H2AX (*γ*-H2AX) foci per cell by 8 Gy X-ray irradiation after 0.5, 1, 4, 8, 16, and 24 hours postradiation (Figures 1(c) and 1(d)). As shown, LSCs contained more *γ*-H2AX foci in nuclei as compared to AECII and AECI cells at 1-hour postradiation. In addition, we observed that the number of *γ*-H2AX foci was increased more than 8-fold in LSCs and only 3-4-fold in AECII and AECI cells relative to unirradiated control (Figure 1(d)). Furthermore, the foci numbers of *γ*-H2AX were increased rapidly and significantly in each cell type following radiation (0.5 hour), but with time, the numbers returned to baseline controls by 16 or 24 hours indicating the repair of DNA damage (Figure 1(d)). It is noted that after 16 hours, the residual foci in LSCs and AECII cells had returned to baseline (Figures 1(c) and 1(d)), but the *γ*-H2AX-positive foci in AECI cells were still detectable, suggesting that the repair of DNA double-strand breaks was delayed (Figures 1(c) and 1(d)). These results indicate that the DNA repair ability in LSCs and AECII cells was more efficient compared with that observed in AECI cells up to 16 hours postirradiation.

At 3-day postirradiation, the morphology of AECII and AECI cells remained unchanged even after high-dose irradiation up to 8 Gy, but the cell size of the LSCs was increased with an average diameter of approximately 5-fold greater than that of the original cells (Figure 1(e)). These cellular changes in LSCs resembled the process of lung cell differentiation. To identify the phenotypes of the cells after radiation of LSCs, immunofluorescence analysis was performed using markers for CD157 (LSC marker), SP-C (AECII marker), and T1*α* (AECI marker). A decreased level of CD157 and increased levels of SP-C and T1*α* were observed in LSC samples treated with 8 Gy irradiation (Figure 1(f)). These observations suggest that radiation exposure could influence the proliferation capacity and differentiation of LSCs into alveolar epithelial cells.

### 3.2. Radiation-Induced EMT and Myofibroblast Development in LSCs

**Figure 2:**
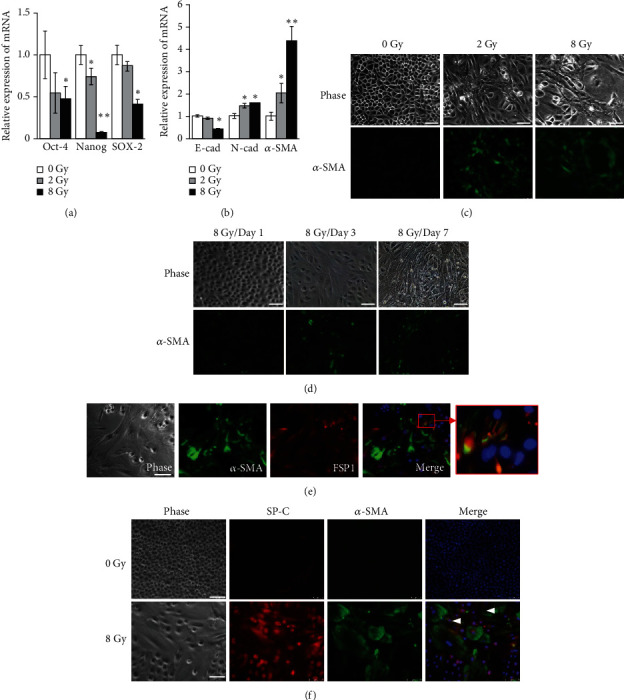
Fibrotic response of LSCs after exposure to irradiation. Total RNA was extracted from irradiated LSCs exposed to 2 or 8 Gy of X-rays followed by incubation under normal conditions for 7 days. The relative levels of the indicated mRNA for Oct-4, Nanog, and SOX-2 (a) as well as E-cadherin, N-cadherin, and *α*-SMA (b) were measured by real-time PCR. (c, d) The represented *α*-SMA expression in irradiated LSCs exposed to 2 or 8 Gy of X-ray followed by incubation under normal conditions for 1–7 days. Scale bars, 100 *μ*m. (e) Irradiated LSCs exposed to 8 Gy of X-ray followed by incubation under normal conditions for 7 days. Cells were double stained with *α*-SMA (green) and FSP1 (red) antibodies and then stained with DAPI (blue). Scale bars, 100 *μ*m. (f) LSCs were double stained with pro-SP-C (red) and *α*-SMA (green) antibodies and then stained with DAPI (blue). The white arrows indicate SP-C^+^/*α*-SMA^+^ cells. Scale bars, 100 *μ*m.

As the time after exposure may account for the differences for radiation effects in lung tissue, such as pneumonitis and fibrosis, the long-term effects of irradiated-LSCs were explored by analyzing fibrogenic differentiation. Recent studies have indicated that EMT may be involved in the process of epithelial cells undergoing phenotypic changes and contribute to the development of fibrosis. Therefore, the mRNA and protein expression profiles of EMT- and myofibroblast-associated genes in LSCs were examined at 7-day postirradiation (Figures 2(a)–2(e)). Compared to control LSCs (0 Gy), the expressions of stem cell markers (Oct4, Nanog, and SOX-2) in irradiated LSCs were decreased in a dose-dependent manner on day 7 (Figure 2(a)). A decreased expression in the epithelial marker E-cadherin was observed in LSCs exposed to 8 Gy, while increased expression of mesenchymal marker N-cadherin was found in LSCs exposed to 2 or 8 Gy, indicative of EMT (Figure 2(b)). In addition, the level of myofibroblast marker, *α*-SMA expression, was significantly increased in irradiated LSCs (Figure 2(b)). Next, we determined whether the increase in *α*-SMA transcript correlated with the increased expression of protein. After exposure to serial doses of radiation at day 7, we found that the *α*-SMA^+^ cells in the irradiated LSCs were more than that in control LSCs (0 Gy) (Figure 2(c)). In addition, the morphological changes in LSCs and the increasing population of *α*-SMA^+^ cells can be induced by radiation exposure in the first 3 days and would last to the 7th day (Figure 2(d)). The enlarged irradiated LSCs coexpressed *α*-SMA and the fibroblast marker FSP1 (Figure 2(e)). In contrast, the majority of LSCs that differentiated into AECII cells, which were characterized by SP-C immunostaining, did not express *α*-SMA (Figure 2(f)). Specifically, a small population of *α*-SMA^+^ and SP-C^+^ irradiated LSCs was found (Figure 2(f), arrow), which implies that LSCs differentiating into AECII cells undergo EMT and develop fibrosis after irradiation.

### 3.3. Isolation and Characterization of LSCs from Neonatal Mice following Exposure to Radiation

**Figure 3:**
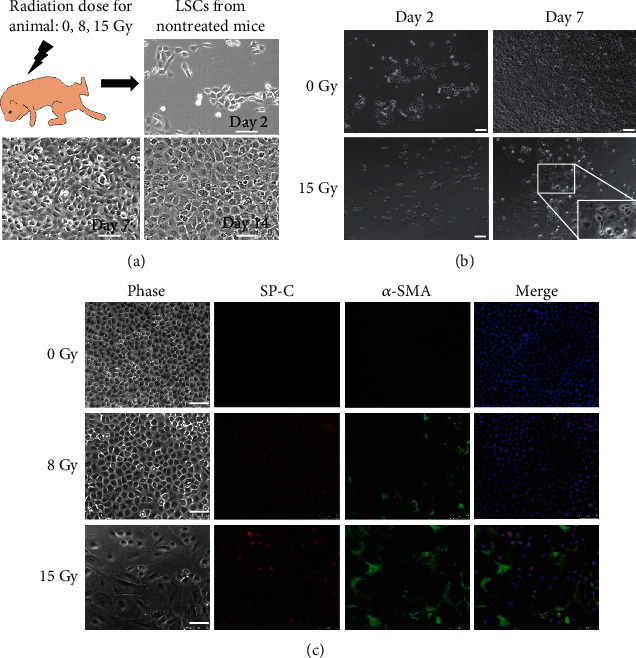
The fibrogenic differentiation of LSCs from irradiated mice. (a) Workflow of *ex vivo* lung cell irradiation experiments. The cell growth and differentiation of LSCs isolated from irradiated neonatal mice were analyzed following incubation for 14 days and compared with those isolated from nontreated mice. Scale bars, 100 *μ*m. (b) LSCs were isolated from control neonatal mice or mice exposed to the indicated dosage of radiation. Cell morphology and density were examined after seeding 1 × 10^5^ isolated LSCs within 5 days. Scale bars, 100 *μ*m. (c) The populations of *α*-SMA^+^ (green) and SP-C^+^ (red) cells were costained in isolated LSCs and were analyzed, followed by incubation for 14 days. The nuclei of cells were stained blue with DAPI. Scale bars, 70 *μ*m.

To determine the effects of radiation exposure on LSCs *ex vivo*, the CD45^−^CD54^+^CD157^+^ LSCs were immediately isolated from the lungs of neonatal mice treated with 8 or 15 Gy whole-body irradiation (Figure 3(a)). Radiation exposure did not influence the percentage of CD45^−^CD54^+^CD157^+^ cells (approximately 1.8% to 2.2% of the total population of viable cells) in the lung tissue of irradiated mice, as determined by flow cytometric analysis. To determine the effects of radiation exposure on these isolated LSCs, cell growth and morphology were analyzed within 2-7 days after whole-body exposure to X-rays. At day 2 after primary culture, the isolated LSCs could attach to collagen I-coated plates and maintain colony morphology (Figure 3(b)). A decrease in cell proliferation and morphological changes occurred in isolated LSCs from 15 Gy irradiated mice (Figure 3(b)). After 14 days in culture, the immunostaining analysis revealed that radiation of mice increased *α*-SMA and SP-C expression in a dose-dependent manner for the isolated cells (Figure 3(c)), as compared to the nonirradiated mice. Therefore, these results suggest that radiation treatment in lung tissue altered the isolated LSCs in epithelial proliferative capacity and fibrotic differentiation in a dose-dependent manner *ex vivo*.

### 3.4. Radiation Exposure Alters Expression of Several Fibrosis-Associated Genes

**Figure 4:**
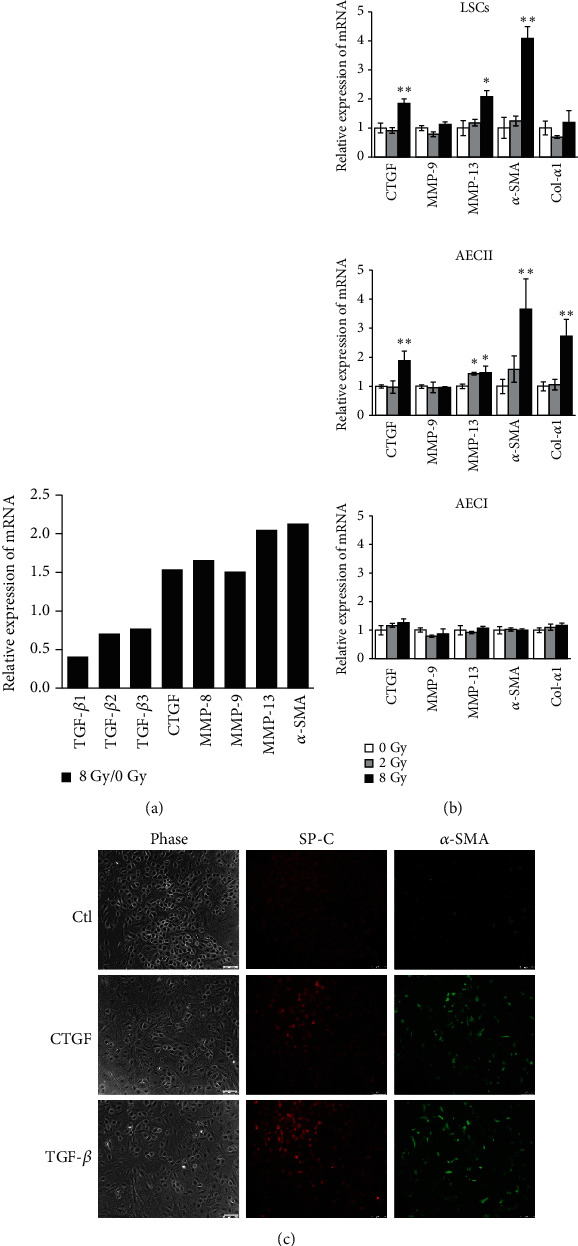
Radiation exposure promotes fibrogenic signaling in LSCs and AECII cells. (a) The mRNA was extracted from LSCs after treatment with or without 8 Gy X-ray followed by incubation under normal conditions for 7 days. Relative gene expression was examined by the Fibrosis PCR Array. (b) The mRNA was extracted from LSCs, AECII, and AECI cells exposed to 2 or 8 Gy. The relative levels of the indicated mRNAs were measured by real-time PCR. The data were normalized to GAPDH and expressed relative to nonradiated control cells. (c) LSCs were treated with CTGF (50 ng/ml) or TGF-*β* (5 ng/ml) for 3 days, and the populations of *α*-SMA^+^ and SP-C^+^ cells were analyzed. Scale bars, 100 *μ*m.

Since the upregulation of *α*-SMA found in isolated LSCs after whole-body X-ray exposure *ex vivo* was similar to that observed in *in vitro* irradiation, a mouse fibrosis PCR array of total RNA samples from LSCs at day 7 postirradiation was performed to uncover the mechanism contributing to fibrosis development in LSCs. Compared to nonirradiated controls, the expressions of TGF-*β* family members (TGF-*β*1, TGF-*β*2, and TGF-*β*3) were decreased, but the expressions of CTGF and matrix metallopeptidases (MMPs) were upregulated in irradiated LSCs (Figure 4(a)). CTGF had been reported to modulate many signaling pathways responsible for tissue remodeling and fibrosis development [17]. MMPs are a family of proteolytic enzymes involved in the degradation and remodeling of extracellular matrix proteins. Therefore, the expressions of CTGF, MMPs, *α*-SMA, and collagen I (Col-*α*1) in irradiated LSCs and alveolar cells were examined by Q-PCR analysis (Figure 4(b)). As shown, the mRNA expressions of CTGF, *α*-SMA, and MMP-13 were upregulated in LSCs and AECII cells after 8 Gy irradiation. However, no significant change in the expression of these genes was found in the irradiated AECI cells (Figure 4(b)). There was no significant difference in the mRNA expression for MMP-9 in each cell type. Overall, the results supported the hypothesis that radiation exposure in LSCs and AECII cells is responsible for the development of fibrosis.

TGF-*β* and CTGF have been linked to trigger tissue fibrosis and accumulate in the early phases of irradiated lung tissue [18, 19]. The 2-fold increase in CTGF expression after 8 Gy irradiation may reflect changes in the content or activation of multiple cell types, including LSCs and AECII cells (Figure 4(b)). Therefore, we evaluated the effect of CTGF on the regulation of LSC differentiation. As shown in Figure 4(c), after LSCs were exposed to CTGF and TGF-*β*, the protein expressions of *α*-SMA and SP-C were increased within 3 days. These data indicate that radiation induced these secretory regulators released, such as CTGF and TGF-*β*, could contribute to LSC differentiation.

### 3.5. Developing an *In Vitro* Lung Model Platform for Radiation Exposure

**Figure 5:**
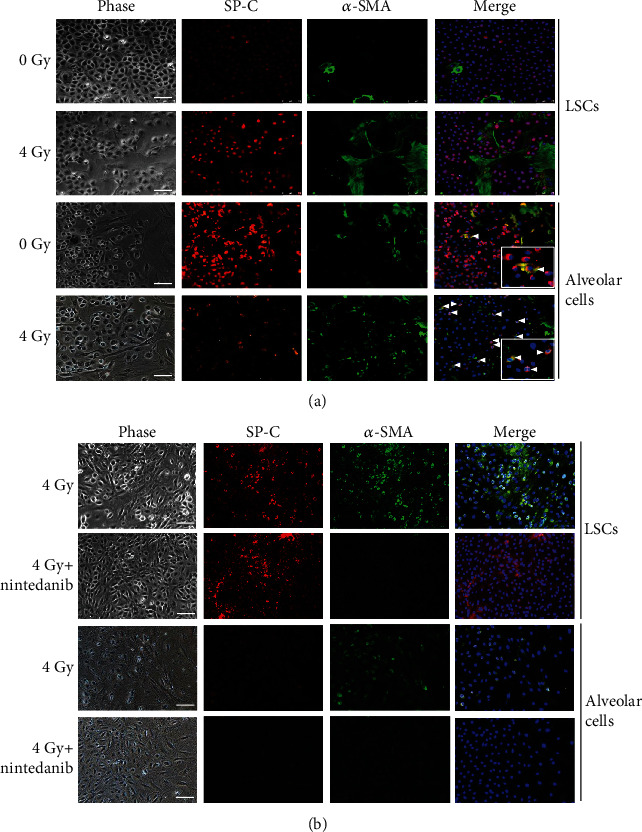
Nintedanib attenuates radiation-induced pulmonary cell fibrogenic differentiation. (a, b) LSCs and LSC-differentiated alveolar cells were exposed to 4 Gy followed by treatment with or without nintedanib (1 *μ*M) and then incubated for 3 days. The levels of SP-C (red) and *α*-SMA (green) protein were determined by coimmunostaining. The arrowheads indicate SP-C^+^/*α*-SMA^+^ cells. The nuclei of cells were stained blue with DAPI. Scale bars, 75 *μ*m.

Based on the differentiation capacity of LSCs as described previously [13, 14], these isolated LSCs and their derived alveolar cells may be suitable to mimic lung tissue *in vivo*. Therefore, the lung differentiation platform was developed to study the effect of radiation exposure in the lung *in vitro*. To construct the *in vitro* lung model platform, LSCs were seeded on culture insert to form an optically confluent monolayer (Supplementary Fig. 1A). A stem cell niche or physiological microenvironment plays a crucial role in the maintenance of stem cell properties. Upon growth factor withdrawal and removing the culture insert, these LSCs were expanded and spontaneously differentiated into alveolar cells (Supplementary Fig. 1). After 5 days of culture, the undifferentiated LSCs located in the central region maintained their phenotypes, while the cells at the edge were migrated out and appeared as thinly spread flattened cell clusters (Supplementary Fig. 1A). Using immunofluorescent analysis, the expressions of alveolar cell markers, SP-C, and T1*α* were found in differentiated cells derived from LSCs (Supplementary Fig. 1B). Therefore, this platform with the LSCs, AECII, and AECI cells was used to examine cell-based responses, pathologic changes, and drug responses in a radiation exposition. After 4 Gy radiation exposure, the undifferentiated LSCs located in the central region differentiated to become either SP-C or *α*-SMA expressing cells (Figure 5(a) first and second panels). However, the expression of *α*-SMA was increased, and SP-C was reduced in the differentiated alveolar cells at the edge region (Figure 5(a) fourth panel). In addition, the increased populations of SP-C^+^/*α*-SMA^+^ cells were observed in the surrounding differentiated cells (Figure 5(a) fourth panel, white arrow). This result indicates that LSCs may differentiate into AECII cells and then undergo either alveolar cell or fibrotic cell differentiation. Therefore, radiation exposure could increase the population of cells that differentiate into myofibroblasts.

### 3.6. Nintedanib Attenuates the Radiation-Induced Fibrogenic Differentiation

Since the LSC, the differentiation platform was capable of mimicking the progression of radiation-induced lung injury, providing a new tool to test cellular responses to drug candidates. Nintedanib, a tyrosine-kinase inhibitor for the treatment of patients with idiopathic pulmonary fibrosis, was used to probe the protective effects for the irradiated LSCs and alveolar cells. The level of *α*-SMA but not SP-C was significantly decreased in irradiated LSCs (Figure 5(b) second panel) and alveolar cells (Figure 5(b) fourth panel) treated with nintedanib for 3 days when compared to the irradiated control cells. These results demonstrate a partial effect on the fibrogenic differentiation of the irradiated LSCs and alveolar cells by nintedanib but not on the formation of alveolar epithelial cells.

## 4. Discussion

**Figure 6:**
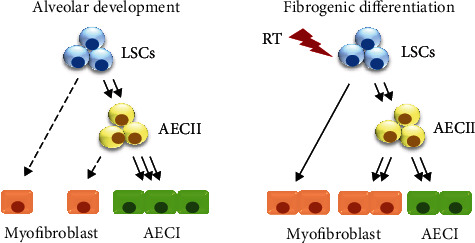
Model of LSC differentiation in alveolar development and irradiated tissue. LSCs (blue): lung stem cells; AECII (yellow): type II alveolar epithelial cells; AECI (green): type I alveolar epithelial cells; RT: radiation.

Severe radiation-induced lung damage, including pneumonitis and fibrosis, remains the major clinical complication of radiotherapy in patients with thoracic malignancies such as lung cancer and esophageal cancer. Advanced methods, such as intensity-modulated radiotherapy and proton therapy, reduce radiation doses to healthy tissues of patients with thoracic cancer [20]. Nevertheless, none of these techniques completely protect lung tissues from radiation effects such as DNA damage. Although all cell types within the same tissue have DNA repair systems that form part of adaptive responses, the relative differences in repair capacity may be correlated with the cell types and developmental stages and can change with time in response to stimuli [21]. LSCs have been shown to induce cell proliferation during the loss of epithelial cells in lung tissue [22], but their role in radiation-induced lung injury is still unknown. Here, we examined the cellular responses of LSCs and their progeny cells when exposed to radiation using stem cell differentiation techniques. Our results showed that radiation exposure was able to promote LSC differentiation into alveolar cells and myofibroblasts. In addition, most of the LSC-derived AECII cells could be further transformed into myofibroblasts following exposure to radiation (Figure 6). Thus, we developed a reliable experimental LSC-based platform that includes alveolar epithelial cells at different stages of differentiation for the study of RILI progression. In addition, this platform may provide a useful tool for radioprotective drug discovery.

Most previous studies were performed to determine the radiation response using immortalized cell line models or radiation-induced lung injury (RILI) animal models [4, 23, 24]. Radiation-induced alveolar epithelial injury followed by abnormal epithelial repair appears to be a key pathological pattern of lung fibrosis. Although the progression of AECII cells and basal cells in lung tissue exposed to radiation has been studied [25, 26], the role of LSCs in RILI is still unclear. Based on their capacity for DNA repair, differentiation potency, and cell proliferation, it is suggested that LSCs may be more beneficial than AECII cells to replenish the alveolar epithelial cells lost in the early stages when lung tissue is exposed to radiation. In this study, we provide a possible role for LSCs in fibrotic development, indicating that they may differentiate into SP-C^+^/*α*-SMA^+^ or *α*-SMA^+^ cells when exposed to radiation. Therefore, LSCs may be a source of the SP-C^+^/*α*-SMA^+^ cells that respond to radiation and increase the risk of organizational defects after radiotherapy. These results are consistent with previous analyses, which showed that *α*-SMA^+^ myofibroblasts can arise from resident epithelial cells that undergo EMT [5, 7, 27]. These SP-C^+^/*α*-SMA^+^ epithelial cells in the lung were also found in patients with chronic obstructive pulmonary disease or idiopathic pulmonary fibrosis [28] and in animals with radiation-induced pulmonary fibrosis [5, 29]. In addition to resident LSCs, exogenous infused epithelial cell types or bone marrow-derived mesenchymal stem cells (MSCs) may modulate tissue injury and repair in regenerative medicine [30]. MSCs are adult stem cells that have the potential to migrate to lesion sites, differentiate into tissue-specific functional cells, and modulate the immunological response, thus enhancing the regeneration of injured tissue. MSCs have been considered for the treatment of RILI, as they can differentiate into lung epithelial and vascular endothelial cells, secrete anti-inflammatory factors (e.g., prostaglandin E2, IL-10, inducible nitric oxide, and indoleamine-2,3-dioxygenase), and restrain the pulmonary epithelial cell EMT process. Induced pluripotent stem cells (iPSCs) can be differentiated into airway cells as a useful tool in the regeneration of whole lung scaffolds after injury [31]. Recently, the treatment efficacy of iPSC-derived conditioned medium in restoring lung epithelial structural damage and suppressing neutrophil infiltration in acute lung injury has been demonstrated [32]. An understanding of the regenerative mechanisms and mediators of MSC- and iPSC-dependent treatment may promote their use in the irradiated lung to facilitate the circulation of stem cells over the damaged tissue.

RILF is characterized by scar formation due to excess wound healing, overproduction, and deposition of ECM proteins and impaired lung function. Tissue engineering approaches using stem cells, growth factors, and biomaterial scaffolds have been employed for regenerative medicine purposes and clinical applications. Therefore, understanding the interactions of stem cells and extracellular microenvironment is required for the design of biomaterials to support the attachment of engineered lung tissues. These above studies based on the cell culture procedure are far from mimicking the *in vivo* lung microenvironment. Three-dimensional scaffolds have been generated from natural and synthetic polymers, including collagen [33, 34], synthetic Matrigel [35], and gelatin [36], to deliver stem cell to the decellularized trachea. Three-dimensional culture of LSCs technology has been performed [37], and these 3D models could be a useful tool to study the mechanism of LSC differentiation involving in radiation-induced fibrosis closer to reality. The cell types of stem cells, implanted materials, and additives introduced above may affect the consequences of transplantation after radiation injury. Therefore, numerous factors need to be taken into consideration, such as the study of tooth restoration, before conducting clinical trials [38].

Cellular processes involved in differentiation required a complex network of several signal pathways activated by multiple growth factors [39]. Besides the various types of stem cells, culture condition may also affect the regenerative and differentiation capacity of stem cells. Recent studies have shown that MSCs can also be cultivated in medium with human platelet lysate instead of FBS, without the loss of their differentiation potential and immunomodulatory effects [40]. Moreover, FBS was substituted with human platelet lysate to generate MSC-derived extracellular vesicles for treating refractory graft versus host disease [41]. Nevertheless, these biological potentials have not been examined in the lung injury model. Even though platelet lysate is more capable of keeping stem cells than FBS, whether platelet lysate can reduce the conversion of LSC into myofibroblasts remains to be further studied.

Stem cell-based platforms are widely used in translational medicine. Human pluripotent stem cells can be used to model disease progression [42], while embryonic cells or adult progenitors with natural characteristics are capable of tissue regeneration or cell lineage tracing in organs [43, 44]. Many lung alveoli model systems have been evolved using organoids grown from lung basal cells or AECII cells [45], but these techniques have rarely been used in radiotherapy research. In the present study, the isolated CD45^−^CD54^+^CD157^+^ LSCs displayed sequential differentiation into alveolar cells and revealed that radiation can trigger alveolar epithelial cells to become alveolar cells and myofibroblasts. In addition, reduced radiation-induced lung fibrosis but not elevated tissue density was reported in the irradiated C57BL6 mice treated with nintedanib [16]. Consistent with these results, our finding using the LSC platform also showed that the nintedanib treatment can attenuate the number of irradiated LSCs and AECII cells undergoing myofibroblast formation but does not affect their differentiation into the alveolar epithelium. While the development of radiation-induced lung injury in an animal model was observed for 4–6 months postradiation, this phenomenon can be detected in the LSC platform within two weeks. In the future, the LSC platform could be used to unravel the cell control mechanisms or to develop radioprotective agents.

Glucocorticoid therapies are unlikely to improve the health of patients with RILI. These therapies include experimental agents (nintedanib plus prednisone, pirfenidone, etc.) and MSCs, which have been used in clinical trials as therapeutics and are becoming increasingly important for alleviating irradiated tissue damage. To date, no significant improvement has been found in clinical trials of MSC-based therapy with anti-inflammatory agents and antibiotics for RILI [46]. Although nintedanib treatment attenuates tissue fibrosis after RILI [16], our results show that nintedanib used on irradiated alveolar epithelium was not beneficial for the attenuation of regenerative lung cells. A combination of stem cell therapy and prescription drugs to retain tissue regeneration may be a potential treatment for RILI.

## 5. Conclusions

Our study revealed that LSCs are sensitive to radiation-induced cell damage and may be involved in radiation-induced lung fibrosis. The LSC platform may contribute to the *in vitro* investigation of lung injury pathogenesis and can be applied to the development of future therapies.

## Data Availability

All data used to support the findings of this study are included within the article.

## Supplementary Materials

Supplementary Materials Supplementary Figure 1: identification of cell differentiation in lung differentiation platform. Supplementary Table 1: primers used in quantitative real-time PCR analysis.
