# Supplementary material for: Radiation Induces Pulmonary Fibrosis by Promoting the Fibrogenic Differentiation of Alveolar Stem Cells
Source: Stem Cells Int. 2020 Sep 29;2020:6312053. doi: 10.1155/2020/6312053 (PMC7542528; doi:10.1155/2020/6312053)
Supplement: Supplementary Materials — Supplementary Figure 1: identification of cell differentiation in lung differentiation platform. Supplementary Table 1: primers used in quantitative real-time PCR analysis. [file 6312053.f1.docx]

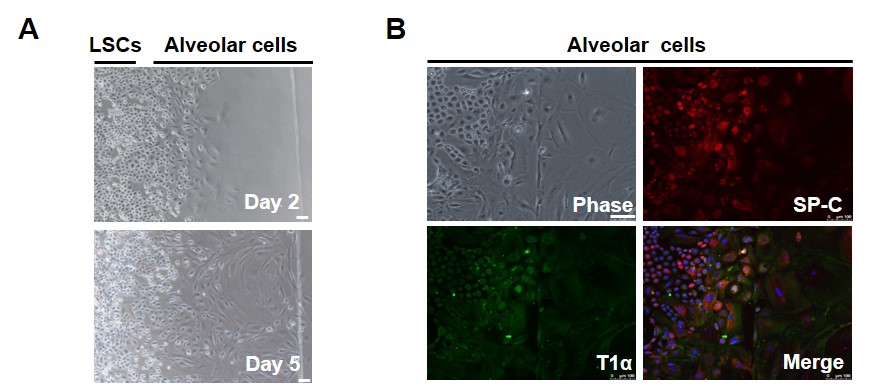


**Supplementary figure 1. Identification of cell differentiation in lung differentiation platform.**

(A)The isolated LSCs were seeded in culture insert and incubated for 5 days after culture insert removed. Scale bars, 100 μm. (B) The AECII and the AECI cells were examined in lung differentiation platform at day 5 through co-immunostaining with SP-C (red), T1α (green) antibodies, and DAPI (blue). Scale bars, 100 μm.

**Supplementary table 1: Primers used in quantitative real-time PCR analysis**

| **Gene Name** | **5’ primer designation** | **3’ primer designation** |
| --- | --- | --- |
| E-cadherin | GGT CTC TTG TCC CTT CCA CA | CCT GAC CCA CAC CAA AGT CT |
| N-cadherin | GTG GAG GCT TCT GGT GAA AT | CTG CTG GCT CGC TGC TT |
| Oct4 | GCA TTC AAA CTG AGG CAC CA | AGC TTC TTT CCC CAT CCC A |
| Nanog | AGG GTC TGC TAC TGA GAT GCT CTG | CAA CCA CTG GTT TTT CTG CCA CCG |
| Sox4 | GAG CGG AGA AAA ATC ATG GA | CGA GGC CGG TAC TTG TAG TC |
| CTGF | GCA AGG AGT GGG TGT GTG | TGT GTC TTC CAG TCG GTA GG |
| MMP-9 | AAC ATC TGG CAC TCC ACA CC | GCA GAA GTT CTT TGG CCT GC |
| MMP-13 | AGA CTG GTA ATG GCA TCA AGG | GCC ATT TCA TGC TTC CTG ATG |
| α-SMA | AGC CAT CTT TCA TTG GGA TG | TAC CCC CTG ACA GGA CGT TG |
| Collagen-αI | CCA AGG GTA ACA GCG GTG AA | CCT CGT TTT CCT TCT TCT CCG |
| GADPH | TGC CCC CAT GTT TGT GAT G | TGT GGT CAT GAG CCC TTC C |
